# A Systematic Strategy to Find Potential Therapeutic Targets for *Pseudomonas aeruginosa* Using Integrated Computational Models

**DOI:** 10.3389/fmolb.2021.728129

**Published:** 2021-09-20

**Authors:** Fernando Medeiros Filho, Ana Paula Barbosa do Nascimento, Maiana de Oliveira Cerqueira e Costa, Thiago Castanheira Merigueti, Marcio Argollo de Menezes, Marisa Fabiana Nicolás, Marcelo Trindade dos Santos, Ana Paula D’Alincourt Carvalho-Assef, Fabrício Alves Barbosa da Silva

**Affiliations:** ^1^ Programa de Computação Científica, Fundação Oswaldo Cruz, Rio de Janeiro, Brazil; ^2^ Laboratório Nacional de Computação Científica, Petrópolis, Brazil; ^3^ Instituto de Física, Universidade Federal Fluminense, Niterói, Brazil; ^4^ Laboratório de Pesquisa Em Infecção Hospitalar, Instituto Oswaldo Cruz, Fundação Oswaldo Cruz, Rio de Janeiro, Brazil

**Keywords:** Pseudomonas aeruginosa, metabolic network, transcriptome data, integrated model, therapeutic target

## Abstract

*Pseudomonas aeruginosa* is an opportunistic human pathogen that has been a constant global health problem due to its ability to cause infection at different body sites and its resistance to a broad spectrum of clinically available antibiotics. The World Health Organization classified multidrug-resistant *Pseudomonas aeruginosa* among the top-ranked organisms that require urgent research and development of effective therapeutic options. Several approaches have been taken to achieve these goals, but they all depend on discovering potential drug targets. The large amount of data obtained from sequencing technologies has been used to create computational models of organisms, which provide a powerful tool for better understanding their biological behavior. In the present work, we applied a method to integrate transcriptome data with genome-scale metabolic networks of *Pseudomonas aeruginosa*. We submitted both metabolic and integrated models to dynamic simulations and compared their performance with published *in vitro* growth curves. In addition, we used these models to identify potential therapeutic targets and compared the results to analyze the assumption that computational models enriched with biological measurements can provide more selective and (or) specific predictions. Our results demonstrate that dynamic simulations from integrated models result in more accurate growth curves and flux distribution more coherent with biological observations. Moreover, identifying drug targets from integrated models is more selective as the predicted genes were a subset of those found in the metabolic models. Our analysis resulted in the identification of 26 non-host homologous targets. Among them, we highlighted five top-ranked genes based on lesser conservation with the human microbiome. Overall, some of the genes identified in this work have already been proposed by different approaches and (or) are already investigated as targets to antimicrobial compounds, reinforcing the benefit of using integrated models as a starting point to selecting biologically relevant therapeutic targets.

## Introduction

Infectious diseases are a concerning public health problem worldwide. Among the most life-threatening infectious diseases are the bacterial infections caused by the “ESKAPE” pathogens, an acronym for *Enterococcus faecium*, *Staphylococcus aureus*, *Klebsiella pneumoniae*, *Acinetobacter baumannii*, *Pseudomonas aeruginosa*, and *Enterobacter* spp., which are known for their ability to escape the action of multiple drugs (Pachori, Gothalwal, and Gandhi 2019). As result, the World Health Organization classified some of these multidrug-resistant pathogens like *P. aeruginosa* as a critical priority on the pathogens list for new antibiotics research and development (World Health Organization 2017). *P. aeruginosa* is an opportunistic human pathogen known for its metabolic versatility, virulence factor diversity, and great intrinsic and acquired antibiotic resistance. These traits allow the bacterium to cause infections in different areas, e.g., the lower respiratory tract, skin, urinary tract, eyes, leading to bacteremia, endocarditis, and other complications (Lister, Wolter, and Hanson 2009; Silby et al., 2011).

Identifying potential targets for new drug discovery or drug repurposing is achieved when crucial biological processes are well-characterized. The complete genome of *P. aeruginosa* PAO1, widely used as a reference strain, was sequenced two decades ago by Stover et al. (2000). Furthermore, the ongoing advance of “omics” technologies had provided even more details to unveil the functioning of *P. aeruginosa*. From a systems biology perspective, these data are the fundamentals of different computational approaches such as the reconstruction of biological networks, a mathematical representation of cell molecules and their interactions. The most common types of biological networks are the metabolic, gene regulatory, and signaling networks (Koutrouli et al., 2020). Genome-scale metabolic networks (GEMs) encompass a set of known biochemical reactions of an organism using gene-protein-reaction (GPR) associations, constrained in some models by thermodynamic directionality, transcription factor activity, gene expression level, and others. The growth rate of an organism in a given condition or the production rate of a metabolite of interest can be predicted from a GEM using optimization methods such as flux balance analysis (FBA) (Ruppin et al., 2010; Hyduke, Lewis, and Palsson 2013).

In addition, we can integrate different types of networks or incorporate additional biological measurements into a single network to provide more robust computational models. Despite the challenge of integrating gene expression data with GEMs, there are several methods proposed to achieve this task. The majority is based on FBA-driven algorithms considering experimentally measured RNA levels to turn off or to constrain the reactions, such as PROM, E-Flux, CoRegFlux, TRFBA, and others (Blazier and Papin 2012; Banos, Trébulle, and Elati 2017; Cruz et al., 2020). PROM is one of the first methods developed to be applied to genome-scale networks in an automated manner. PROM uses transcriptome data to define gene activation or repression, and interactions between regulators and targets. However, it requires a large amount of data (Chandrasekaran and Price 2010). E-Flux defines the reaction maximum flux to the gene expression level, while CoRegFlux applies a statistical approach to infer the gene regulatory network from transcriptome data (Colijn et al., 2009; Banos, Trébulle, and Elati 2017). TRFBA uses the gene expression level converted by the constant parameter *C* to constrain the reaction upper bound. TRFBA does not require a large amount of data nor previous knowledge of the regulator-target relationship (Motamedian et al., 2017; Malek Shahkouhi and Motamedian 2020).

The growth rate assessment using biological networks enables the prediction of drug targets since gene knockout can result in growth arrest or growth loss. Likewise, the cell response to antimicrobial compounds can be predicted (Chavali et al., 2012; Chung et al., 2021). Indeed, this approach was applied to several pathogens, including ESKAPE Gram-negative bacteria as *A. baumannii*, *K. pneumoniae*, and *P. aeruginosa*, to identify novel targets and to evaluate the impact of last-resort antibiotics on the metabolism (Presta et al., 2017; Ramos et al., 2018; Zhu et al., 2018; Norsigian et al., 2019). Moreover, it is possible to simulate the temporal and spatial dynamics of the growth process, i.e., in the first step of the simulation, biomass and metabolite production rates calculated using FBA update the extracellular concentrations. In the next step, uptake rates of compounds required for FBA calculation are subjected to the previous updated extracellular concentrations and could lead to environmental changes for the following time step. The process may continue until there are no more substrates available in the extracellular space (Scott et al., 2018).

In this work, we applied the TRFBA method for integrating transcriptome data with two metabolic reconstructions of PAO1. Next, we used the ACBM, an agent and constraint-based modeling approach, to simulate the temporal dynamics of the growth process. The primary goals were: 1) to generate an integrated computational model of *P. aeruginosa*, which incorporates gene expression data in the GEM; and 2) compare the dynamics of metabolic and integrated models to analyze if the progressive inclusion of biological data results in more reliable computational models capable of simulating the biological growth of *P. aeruginosa*. Then, we investigated the hypothesis that identifying potential targets from integrated models is more accurate than from metabolic models that do not consider information from other cellular processes. We used the algorithm FindTargetsWEB (Merigueti et al., 2019) to find these targets from both models and compare them to discuss their accuracy. The goal is to show improved selectivity and (or) specificity of target prediction from integrated models compared to metabolic models.

## Materials and Methods

### Data Selection

The genome-scale metabolic models of *P. aeruginosa* PAO1 used in this work were iMO1056 and iPAO1 (Oberhardt et al., 2008; Zhu et al., 2018). The iMO1056 model contains 992 reactions, 858 metabolites, and 1,042 genes encompassed in the cytoplasmic and extracellular compartments. This metabolic model is the first genome-scale metabolic model of *P. aeruginosa*, also used extensively in the literature. The iPAO1 model contains 4,365 reactions, 3,022 metabolites, and 1,458 genes encompassed in the cytoplasmic, periplasmic, and extracellular compartments. It is the only model of *P. aeruginosa* that includes the periplasmic space. We edited the iMO1056 model by adding calcium and chloride ions into the biomass reaction, and their corresponding exchange and transport reactions, because they are essential molecules to the cellular homeostasis and components of the growth media used in our work. Based on biological measurements obtained from *Pseudomonas* genus, we also adjusted the non-growth-associated maintenance value to 3.96 mmol ATP gDW^−1^ h^−1^ in both models (van Duuren et al., 2013). The transcriptome data used in this work are available under the access number E-MTAB-8374 at Array Express database and was performed by Dolan et al. (2020), where total RNA was isolated from cells grown in MOPS minimal medium supplemented with glycerol or acetate as the carbon source. Furthermore, to reproduce these same biological conditions, we properly adjusted the lower and upper bounds of exchange reactions in both computational models.

### Transcriptome Data Analysis

First, we assessed the raw reads of RNA-sequencing experiment in fastq format using FastQC (Andrews 2010). Then, we performed quality and adapter filtering using Trimmomatic with default parameters when necessary (Bolger, Lohse, and Usadel 2014). For the alignment of the processed reads to the PAO1 genome (available at GenBank database under the accession number NC_002516), we used HISAT2 (version 2.2.0) (Kim et al., 2019). We used the featureCounts program to count the number of reads mapped to each coding sequence of the PAO1 genome (Liao, Smyth, and Shi 2014) and we normalized the transcript abundance by applying the transcripts per million measure (Wagner, Kin, and Lynch 2012).

### Integration of Metabolic Network and Transcriptome Data

We used the TRFBA algorithm in its linear form, i.e. the version which integrates a metabolic network with expression data to model a specific condition, to construct the integrated models of PAO1 from iMO1056 and iPAO1 models, and the chosen transcriptome data (Motamedian et al., 2017; Malek Shahkouhi and Motamedian 2020). First, the TRFBA converts all reactions in the model to their irreversible form. Given a reversible reaction R:A⇄B, the algorithm splits R into $R_{1}:A\to B$ and $R_{2}:B\to A$; thus, each direction of R is written in its irreversible form. For the following steps, the integration process requires GPR statements. GPR is the association between the enzyme(s) that catalyzes the reaction and the gene(s) that codifies this (ese) enzyme(s). If the GPR contains enzymes that catalyze the same reaction but are coded by different genes (isozymes), the expression of either protein is required to the catalysis, and the GPR is represented with the logic operator *OR*. Given a reaction *R* associated with isoenzymes coded by different genes g, the algorithm replicates *R* as $\left\{R_{g_{1}},\;\ldots ,\;R_{g_{n}}\right\}$, where n is the number of isozymes. Thus, each isozyme is individually associated with one copy of R. If the reaction is catalyzed by an enzyme composed of subunits coded by different genes, the expression of all subunits is required and the GPR is represented with *AND*. Therefore, no modifications are required. After these rules are applied, the upper bound of all reactions in the model is constrained according to the lower expression level among all associated genes (limiting rate) multiplied by the constant parameter *C*, representing the maximum rate supported by one unit of a gene expression level.

### Flux Balance and Variability Analysis

FBA is a constraint-based mathematical approach used to calculate the fluxes of a metabolic network under the steady-state by optimizing an objective function through linear programming (Orth, Thiele, and Palsson 2010). In this work, we set growth rate as the objective function to be maximized. As the optimal growth rate is calculated, FBA returns a single flux distribution. However, different flux distributions are possible for the same maximal growth. Flux variability analysis (FVA) is a mathematical approach used to determine the minimum and maximum flux value for each reaction in a given model obeying the same constraints and the same objective value as FBA within the solution space (Mahadevan and Schilling 2003; Schellenberger et al., 2011). We used the functions optimizeCbModel (FBA) e fluxVariability (FVA) from COBRA Toolbox to perform these analyses.

### Dynamic Simulations of Metabolic and Integrated Models

We used the user-friendly ACBM framework to simulate the growth of *P. aeruginosa* PAO1 over time using both metabolic and integrated models (Karimian and Motamedian 2020). Briefly, it uses agent and constraint-based modeling to apply intracellular and extracellular restrictions to the cell population in a three-dimensional space, where each cell, metabolite (carbon source), and environment are modeled as an agent. ACBM requires the input of several parameters such as initial cell amount, radius, length, mass, initial metabolite amount, and volume of the simulated environment. The movement of metabolites and cells in the environment is predicted based on stochastic simulations and FBA or TRFBA are used to predict growth. In each time step, if there is a metabolite close to the cell, it consumes the metabolite, moves at random, and the metabolite is removed from the environment. If there is no metabolite, the cell moves at random. All possible events, such as biomass generation, production, or consumption of metabolites are calculated and updated to the next step. If the cell mass doubles, a new cell object is included in the environment (cell division). Cells are removed from the environment when they do not find metabolites during their survival time (cell death). This process continues until the simulation time ends or until all cells die. The computational models and parameters given as input to ACBM (Supplementary Table 1: https://github.com/medeirosfilho1/Integration-paeruginosa/blob/main/Supplementary_table_1.xlsx) were based on experimental data obtained in previous studies (Dolan et al., 2020; H. ; Zhang et al., 2016).

### Identification of Potential Therapeutic Targets

We used the FindTargetsWEB online application to identify potential therapeutic targets in PAO1 metabolic and integrated models (Merigueti et al., 2019). The same models used to perform dynamic simulations were converted from MAT to SBML level 3 format using the writeSBML function from COBRA Toolbox, which is the file format required by FindTargetsWEB (all files are available at: https://github.com/medeirosfilho1/Integration-paeruginosa). The application is composed of nine steps. The first step evaluates if the model generates a biomass value greater than zero. As a second step, FindTargetsWEB uses FVA to filter reactions whose flux range is equal to zero. Since those reactions do not accept any variation, they may be more susceptible to perturbations (Oberhardt et al., 2010). However, the results of this step are only maintained if the user chooses to perform the FBA + FVA analysis. Therefore, we used the FBA + FVA option for identifying potential targets. The following steps comprise the knockout of single reactions followed by gene knockouts if GPR associations are available. When knockout simulation results in a biomass value of zero, the reaction GPR and (or) gene information is stored. If the knocked-out gene is included in the stored reaction GPR, this gene is considered essential. When GPR associations are not provided, FindTargetsWEB retrieves EC numbers from the KEGG database through the reaction compounds. Otherwise, EC numbers are retrieved through gene ID. Then, EC numbers are used to query the DrugBank database to obtain protein name, organism, and UniProt ID. The UniProt ID is used to perform blastp searches against the genome of the model organism. Proteins with identity ≥30% are kept. Finally, FindtargetsWEB uses the recovered UniProt IDs to search for inhibitors in the DrugBank database. After obtaining the results from FindTargetsWEB, we filtered the application output to keep only hits with identity ≥60% and coverage ≥70% and used these proteins to carry out the analysis to prioritize targets according to non-host homology and microbiome conservation. First, we performed a blastp search against the human proteome (GRCh38. p13 release available at RefSeq database under the accession number GCF_000001405.39) using E value ≤ 1e-5 and coverage ≥70% as parameters. Next, hits with identity ≥40% were filtered out. Likewise, the remaining proteins were compared to the proteome of 454 organisms (Supplementary Table 2: https://github.com/medeirosfilho1/Integration-paeruginosa/blob/main/Supplementary_table_2.xlsx) from the gastrointestinal tract of NIH Human Microbiome Project (Human Microbiome Project Consortium 2012a; 2012b). Alignments with identity ≥40% were considered as a hit.

### Technical Specifications

TRFBA algorithm, ACBM framework, FBA, and FVA analysis were performed using the MATLAB program (R2020b version 9.9) with the COBRA Toolbox (version 2.0.0) and the gplk solver (version 2.7) in the Java runtime environment (version 1.8.0_281).

## Results

### Dynamic Simulations of Metabolic and Integrated Models

**FIGURE 1:**
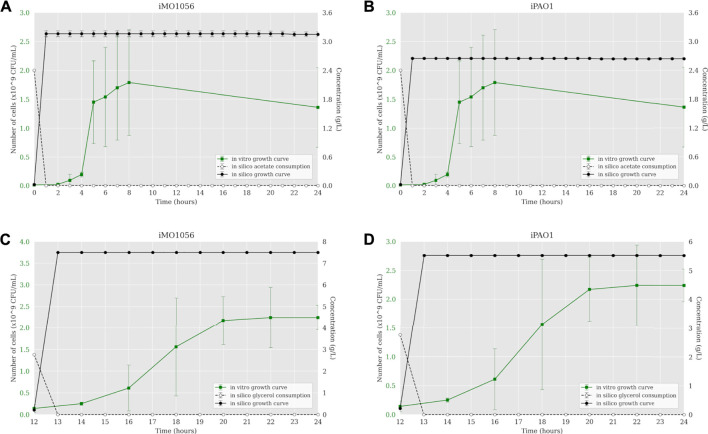
Comparison between *P. aeruginosa* growth curves predicted by ACBM from the metabolic models and growth curves measured by Dolan et al. (2020). **(A,B)** MOPS minimal medium with acetate as carbon source. **(C,D)** MOPS minimal medium with glycerol as carbon source. The graphics include the carbon source consumption over time (dashed lines). All measurements were made at least in triplicates, and curves represent the mean of all replicates. Error bars are the standard deviation of the mean.

The iMO1056 and iPAO1 metabolic models were modified to reproduce the experimental environment used by Dolan et al. (2020). First, we adjusted the lower and upper bounds of all exchange reactions to zero except those related to the MOPS minimal medium compounds and carbon source, which were set to −1,000 (lower bound) and 1,000 (upper bound). Then, we performed the FBA analysis of metabolic models. The iMO1056 model generated a biomass reaction flux of 11.26 h^−1^ for acetate as a carbon source and 21.90 h^−1^ for glycerol as a carbon source. The iPAO1 model generated a biomass reaction flux of 8.68 h^−1^ and 17.32 h^−1^ for acetate and glycerol, respectively. After, the ACBM framework was used to dynamically simulate *P. aeruginosa* PAO1 growth in MOPS minimal medium with acetate (Figures 1A,B) or glycerol (Figures 1C,D) as a carbon source. Since ACBM executions are computationally expensive, the environment volume simulated is only 0.16 μL. Therefore, due to the randomness and discretization effects, it would be improper to directly compare experimental results generated in a counting unit (CFU/mL) and computational results generated in a concentration unit (g/L), whereas the numbers obtained during simulations are relatively low. Instead, we choose to compare the exponential phase duration, entry points in the stationary phase, and growth curve shapes. Simulations revealed that the growth curve reached the stationary phase after 1 hour. In the same way, the carbon sources were consumed in less than 1 hour.

**FIGURE 2:**
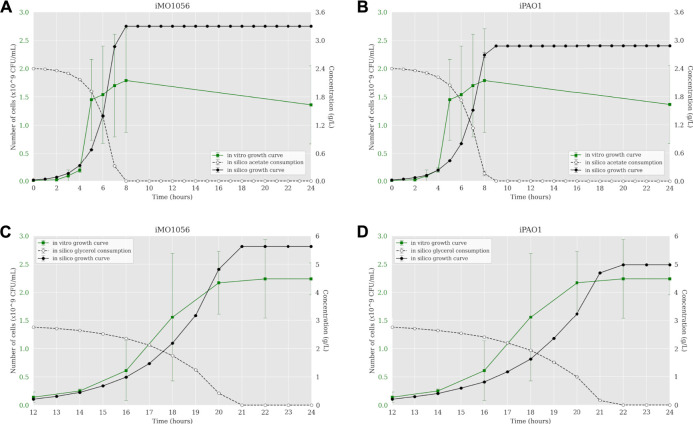
Comparison between *P. aeruginosa* growth curves predicted by ACBM from the metabolic models with adjusted carbon source uptake rates and growth curves measured by Dolan et al. (2020). **(A,B)** MOPS minimal medium with acetate as carbon source. **(C,D)** MOPS minimal medium with glycerol as carbon source. The graphics include the carbon source consumption over time (dashed lines). All measurements were made at least in triplicates, and curves represent the mean of all replicates. Error bars are the standard deviation of the mean.

The next step was to insert experimentally measured uptake rates for both carbon sources as input to the ACBM framework. The default uptake upper bound value is 1,000 mmol gDW^−1^ h^−1^. In line with Dolan et al. (2020), we adjusted this parameter to 30.4 mmol gDW^−1^ h^−1^ for acetate and 9.2 mmol∙gDW^−1^ h^−1^ for glycerol. Figure 2 shows a predicted behavior closer to the observed biologically depicting a sigmoidal curve typical of bacterial growth. When acetate was the carbon source, cells reached the stationary phase after 7 and 8 h of simulation from the iMO1056 and iPAO1 models, respectively, almost depleting acetate concentration (Figures 2A,B). When glycerol was the carbon source, cells reached the stationary phase at 21 and 22 h of simulation from the iMO1056 and iPAO1 models respectively, almost depleting glycerol concentration (Figures 2C,D).

**FIGURE 3:**
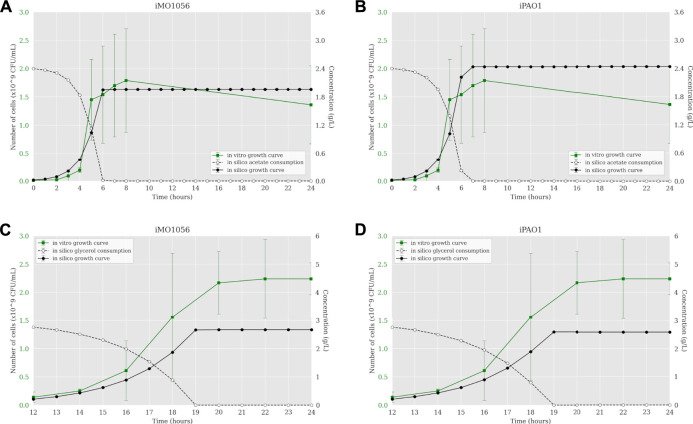
Comparison between *P. aeruginosa* growth curves predicted by ACBM from the integrated models and growth curves measured by Dolan et al. (2020). **(A,B)** MOPS minimal medium with acetate as carbon source. **(C,D)** MOPS minimal medium with glycerol as carbon source. The graphics include the carbon source consumption over time (dashed lines). All measurements were made at least in triplicates and curves represent the mean of all replicates. Error bars are the standard deviation of the mean.

According to Motamedian et al. (2017), we calculated the *C* constant value for both metabolic models in each growth condition using the transcriptome data of *P. aeruginosa* PAO1. The resulting *C* values were 0.605 and 0.172 mmol gDW^−1^ h^−1^ in acetate to iMO1056 and iPAO1 models, and 0.063 and 0.043 mmol gDW^−1^ h^−1^ in glycerol to iMO1056 and iPAO1 models respectively. We used these values to perform the integration using the TRFBA algorithm adjusting the growth rates to approximately 0.80 h^−1^ (acetate) and 0.37 h^−1^ (glycerol) as experimentally measured by Dolan et al. (2020). The dynamic simulations with the integrated models predicted growth curves with a shape more similar to the *in vitro* growth curve (Figure 3). In acetate, cells entered the stationary phase after 6 h for both integrated models (Figures 3A,B). Likewise, in glycerol, cells reached the stationary phase at 19 h of simulation (Figures 3C,D).

### Flux Balance and Variability Analysis

**FIGURE 4:**
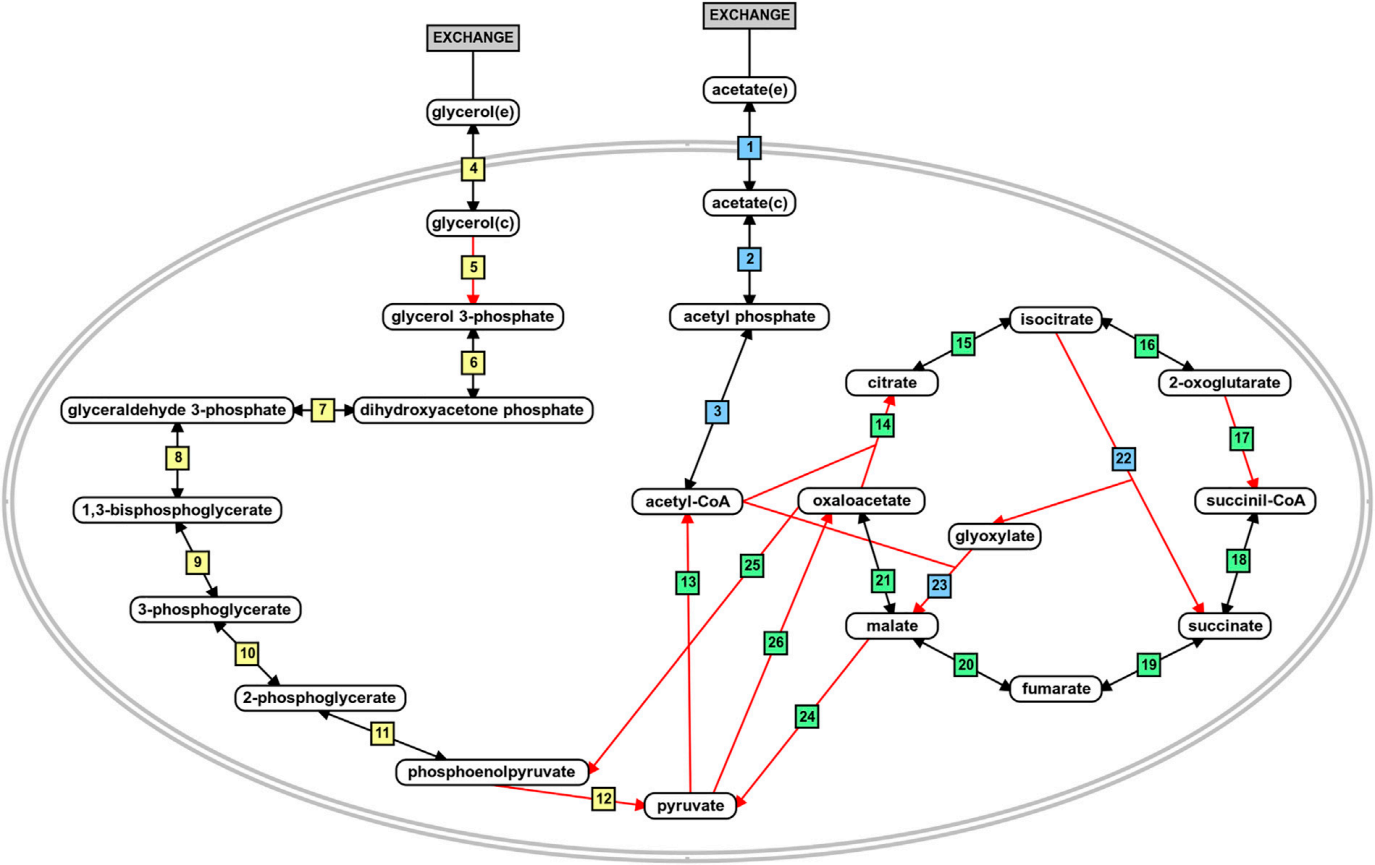
Schematic representation of acetate and glycerol central metabolism in *P. aeruginosa* PAO1. The blue boxes depict reactions related to acetate metabolism. The yellow boxes depict reactions related to glycerol metabolism, and the green boxes are the reactions shared by the metabolism of both carbon sources. The red arrows indicate irreversible reactions. This figure was generated using the PathVisio software (Kutmon et al., 2015).

The flux distribution in the central metabolism of *P. aeruginosa* PAO1 during growth in acetate or glycerol was experimentally measured by Dolan et al. (2020). Based on the results, we analyzed the predicted flux flow through the network upon carbon source uptake for both models in all growth conditions using FBA and FVA. The analysis revealed that the fluxes predicted from the iMO1056 and iPAO1 models with acetate as carbon source agreed with the biological pathway (Figure 4 and Supplementary Table 3: https://github.com/medeirosfilho1/Integration-paeruginosa/blob/main/Supplementary_table_3.xlsx). It is noteworthy that the known utilization of isocitrate by tricarboxylic acid cycle and glyoxylate shunt was computationally reproduced by all models (Figure 4, reaction 15 to reactions 16 and 22). However, the reaction numbered as 21 in Figure 4, although catalyzed by the same enzyme, the malate dehydrogenase, showed a slight difference between metabolic and integrated models. The flux in the metabolic adjusted models was mainly going through the reaction using ubiquinone as a cofactor, while in the integrated models, the flux passed through the reaction with nicotinamide adenine dinucleotide as a cofactor. Regarding the flux values, the predicted acetate uptake rates ranged from 460 to 600 mmol gDW^−1^ h^−1^; consequently, the enchained reactions also had high flux values. FBA analysis from integrated models revealed acetate uptake rates coherent with those experimentally measured ranging from 45 to 52 mmol gDW^−1^ h^−1^.

Likewise, during the simulated growth in glycerol, the uptake rate ranged from 360 to 400 mmol gDW^−1^ h^−1^ in the metabolic models and 13–14 mmol gDW^−1^ h^−1^ in the integrated models. However, the reaction numbered as 5 in Figure 4 of the iPAO1 metabolic models, an essential step to glycerol entrance in the central metabolism, had no flux suggesting the flow was shifted through reaction steps not observed biologically. After integration, the flux through reaction 5 was restored. The flux distribution calculated for all models is available in the Supplementary Material.

### Identification of Potential Therapeutic Targets

**FIGURE 5:**
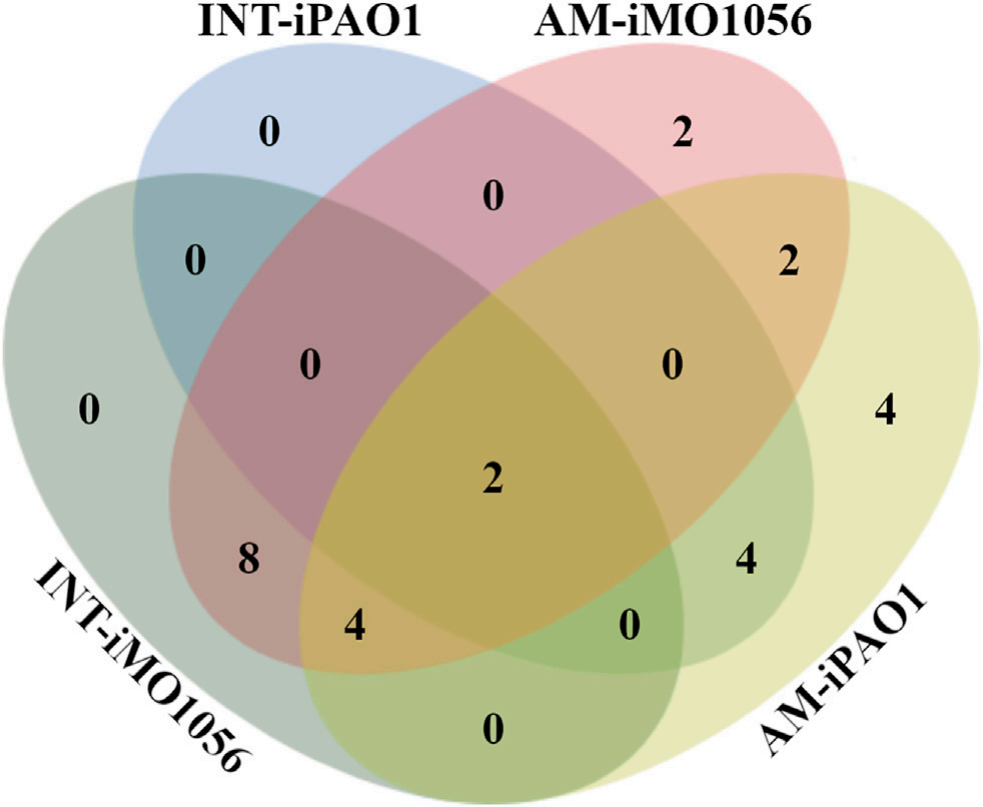
Venn diagram illustrating the number of targets shared by the metabolic models with adjusted carbon source uptake rates (AM) and integrated models (INT).

We used the FindTargetsWEB application (Merigueti et al., 2019) to analyze the selectivity of integrated models in identifying therapeutic targets compared to metabolic models with adjusted carbon source uptake rates, which do not consider gene expression data. Among the eight models analyzed, FindTargetsWEB identified a total of 68 different drug targets. However, the application applies an identity cutoff of 30% to search for similar DrugBank proteins in the target organism. We choose to apply a more conservative filter using an identity greater than 60% and coverage greater than 70%. The number of targets decreased to 32. In addition, to avoid undesirable host-drug interactions, we filtered out *P. aeruginosa* proteins homologous to any human proteins resulting in a final list of 26 targets. Considering both models and no growth condition, 18 of the 26 targets were also predicted from the integrated models, while 8 were unique from adjusted metabolic models (Figure 5 and Table 1).

**TABLE 1:** List of potential therapeutic targets identified by the FindTargetWEB application from the metabolic models with adjusted carbon source uptake rates and integrated models.

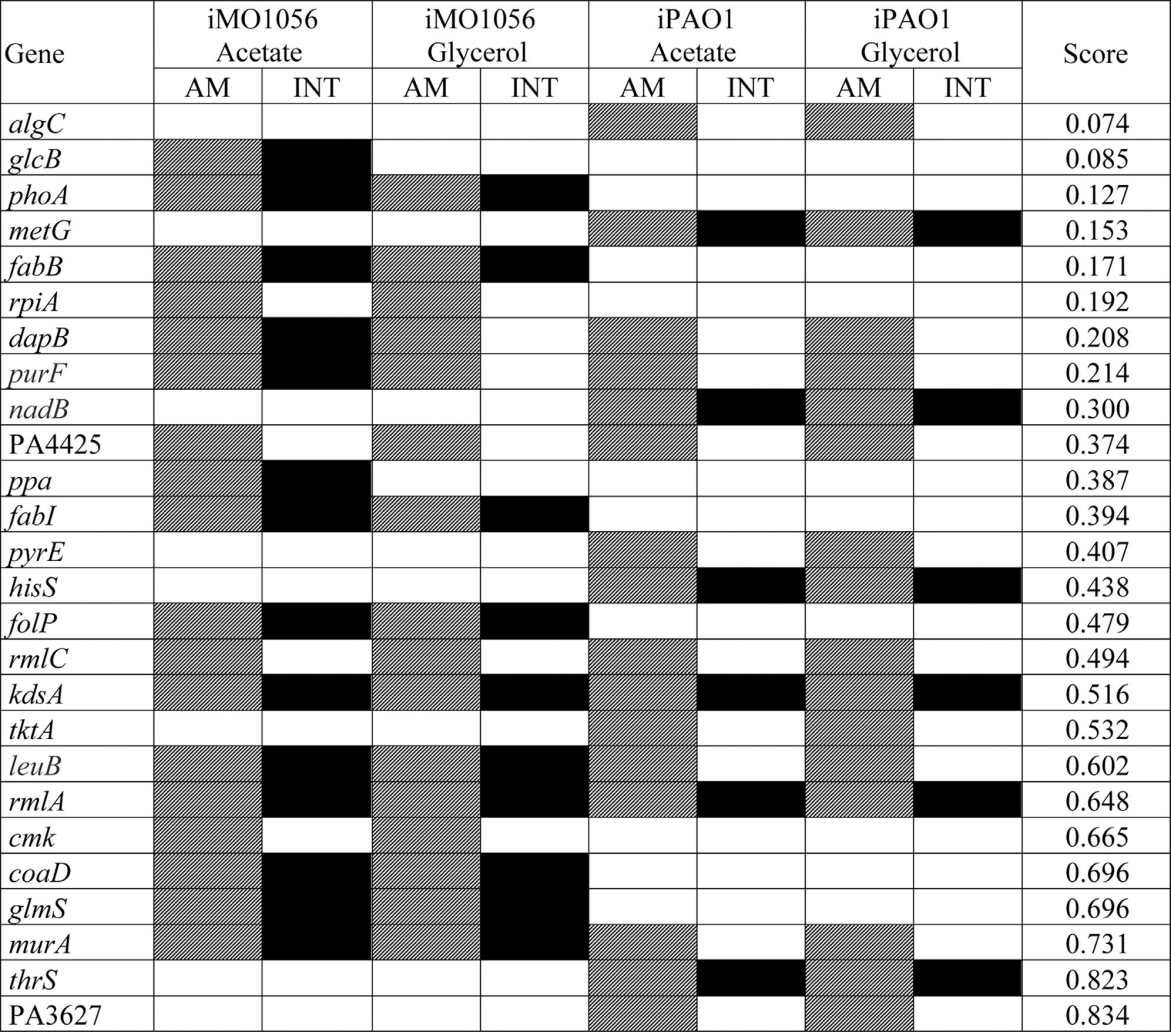
The genes are sorted based on the number of alignments with organisms of the human microbiome in ascending order. The cells filled with diagonal black lines indicate the target was identified from the metabolic models with adjusted carbon sources uptake rate (AM). The cells filled with full black color indicate the target was identified from the integrated models (INT).

Several organisms inhabit the host gastrointestinal tract, and antimicrobial effects upon the normal microbiota could result in adverse effects. Although we do not consider homology an excluding factor, we used blastp results of the predicted targets against the human microbiome to compute a score and suggest a prioritization. The score was the ratio between the number of organisms that presented at least one hit with the target protein sequence and the total number of organisms from the gastrointestinal tract (Table 1). Table 1 emphasizes the selectivity of the integrated models showing the targets predicted by each model in the different growth conditions. It is noteworthy that targets found in integrated models are proper subsets of the targets found in the corresponding metabolic adjusted models. Except for specific genes of the iMO1056 model, there are no differences in the prediction between growth conditions.

## Discussion

Evolving antibiotic resistance profiles emphasize the need to research and develop drug targets and effective therapies against infections caused by *P. aeruginosa*. In the last few decades, computational approaches have become essential tools to help researchers screen new drug targets and hasten drug discovery and design. Reconstruction of biological networks from “omics” data is one of these tools. Moreover, integrating different networks (e.g., metabolic, gene regulatory, signaling networks) is expected to yield more comprehensive computational models that allow a more accurate prediction of any condition of interest. This work aimed to integrate GEMs of *P. aeruginosa* PAO1 with publicly available gene expression data to better reproduce the biological behavior and identify potential therapeutic targets.

The dynamic simulations performed from both metabolic and integrated models have demonstrated that as we include more layers of biological information in the computational model, the more precise the predictions are. According to Dolan et al. (2020), cells grown in MOPS minimal medium with acetate as carbon source show a growth rate of 0.80 h^−1^ and an exponential phase that ranges from 2 up to 8 h. However, the cells grew slower in glycerol (0.37 h^−1^), starting the exponential phase at 12 h of growth and reaching the stationary phase at 20 h. The predictions based on metabolic models show cells reaching the stationary phase before the first hour of simulation, also depleting all carbon sources available (Figure 1). This growth rate is not consistent with biological behavior. Once we adjust carbon source uptake rates to values measured experimentally, the growth curve based on metabolic models showed a more suitable shape. Indeed, in acetate, cells reached stationary phase at closer times than *in vitro* growth, showing a growth rate of 0.82 h^−1^ to iMO1056 and 0.71 h^−1^ to iPAO1 model (Figures 2A,B). Likewise, in glycerol, the shape of the growth curves is less incoherent, showing a growth rate of 0.55 h^−1^ to iMO1056 and 0.48 h^−1^ to the iPAO1 model (Figures 2C,D). These results show that the addition of a single biological measure could improve the accuracy of simulations. The next step was to analyze the dynamics of models after the integration process. Both growth curves had a shape closer to the observed experimentally.

In acetate, cells reached the stationary phase after 6 h of simulation from both models (Figures 3A,B). In glycerol, cells reached the stationary phase at 7 h of simulation from both models, considering the starting point of 12 h, it was equal to 19 h of growth (Figures 3C,D). As part of the integration process, the growth rates of integrated models were equal to those measured by Dolan et al. (2020). However, it is noteworthy that simulations from integrated models showed carbon source uptake rates close to 30.4 (acetate) and 9.2 (glycerol) mmol gDW^−1^ h^−1^. In addition, we analyzed the internal flux distribution. FBA uses linear programming to find reaction fluxes based on maximizing an objective function. In this work, the objective function is biomass production. An optimal solution can obtain non-zero flux values to reactions that are not part of a known biological pathway but are still correct from a mathematical perspective. We observed that sometimes the optimal solution found included fluxes not observed experimentally. However, we could observe that the integration of metabolic networks with transcriptome data allowed the flux to pass through the correct biological pathways. Integrated models have a reduced solution space compared to non-integrated models, which may be related to the flux reorientations observed in the former. These results indicate that the definition of constraints based on expression gene levels allowed the system to approximate to better approximate the experimental growth curve dynamics compared to the previous approaches.

Identification of potential drug targets from metabolic networks is commonly employed. There are different approaches to identify targets from genomic-scale metabolic networks, with several levels of automation. In this work, we used FindTargetsWEB, which is an online application developed to identify targets based on gene essentiality under a given condition. Although metabolic networks are a valuable resource, we could observe that as we include more biological measurements in a computational model, the predictions based on this model are more accurate. Furthermore, since we observed better growth dynamics with integrated models, we suggest identifying targets from these models could also be more reliable. In order to analyze this hypothesis, we submitted both metabolic and integrated models to FindTargetsWEB and compared the results. We found that 18 of the genes identified from the integrated models as potential drug targets were a subset of the 26 genes identified from the metabolic models with adjusted carbon source uptake rates. This observation is a consequence of the solution space reduction due to more strict constraints imposed by the gene expression data. Our intention is not to point out that the genes found from non-adjusted metabolic models are not reliable targets, but the integration is a method to improve selectivity and narrow the screening process.

According to the target scores (Table 1), we highlight the five top-ranked genes selected from the integrated models, *glcB*, *phoA*, *metG*, *fabB*, and *dapB.* Poulsen et al. (2019) pointed out 321 genes of *P. aeruginosa* as high-priority drug targets based on the definition of an essential core genome*.* The essentiality analysis was performed with different strains carrying transposon insertions grown in different media. We observed that the five top-ranked genes are included in the essential core genome described by Poulsen et al. (2019) except for *glcB* and *phoA*. It is noteworthy that the minimal medium used was M9 and the reference strain was PA14 (Poulsen et al., 2019). The fact that the genes listed in Table 1 have already been identified in the literature as potential targets reinforces the adequacy of the method described in our work for selecting biologically relevant targets.

The first placed gene among the five top-ranked is *glcB*, which encodes a malate synthase (MALS). MALS catalyzes the condensation of acetyl-CoA to glyoxylate to form malate and coenzyme A. This reaction is an essential step of the glyoxylate cycle (Figure 4, reaction 23), an anaplerotic pathway providing intermediates for the tricarboxylic acid cycle or precursors for amino acid synthesis. It acts as a modified version of the tricarboxylic acid cycle, bypassing the carbon dioxide-producing steps to conserve carbon atoms for gluconeogenesis (Beeckmans 2009; Kornberg 1966). This glyoxylate shunt is involved in metabolic adaptation to environmental changes, and it is essential for bacterial growth in acetate, ethanol, fatty acids, or any substrate whose acetyl-CoA is a direct product of the pathway. Besides enabling the use of different carbon sources, the glyoxylate shunt plays an important role in virulence, oxidative stress defense, and antibiotic resistance in several clinically relevant pathogens (Maloy, Bohlander, and Nunn 1980; Renilla et al., 2012; Dunn, Ramírez-Trujillo, and Hernández-Lucas 2009; Lorenz and Fink 2002; Meylan et al., 2017). The chronic *P. aeruginosa* infections in cystic fibrosis patients showed MALS upregulation. A double mutant of MALS and isocitrate lyase (another enzyme of the glyoxylate cycle) was avirulent in a mouse pulmonary infection model, which emphasizes MALS as an attractive target for drug development. In addition, MALS has great potential as a broad-spectrum target because it is conserved in pathogenic species, selective, and has a narrow distribution in the gastrointestinal microbiota (Table 1) (Hagins et al., 2010; Fahnoe et al., 2012; Myler and Stacy 2012; Murima, McKinney, and Pethe 2014; McVey et al., 2017). A class of Mg^2+^ chelators compounds called phenyl-diketoacid were described as MALS inhibitors in *Mycobacterium tuberculosis* (Krieger et al., 2012; Shukla, Shukla, and Tripathi 2021). In *P. aeruginosa*, Fahnoe et al. (2012) identified eight different new compounds with inhibitory activities against MALS. Furthermore, these compounds impaired both MALS and isocitrate lyase enzymes within the glyoxylate shunt pathway, an advantageous property to prevent the rapid development of resistance against new antimicrobial agents.

Inorganic phosphate is an essential component of nucleotides, membrane phospholipids, and phosphorylated proteins. In bacteria, phosphonates and organophosphates are viable sources of inorganic phosphate upon the enzymatic activity of alkaline phosphatases (AP). The gene *phoA* encodes a periplasmic AP, and it is highly expressed under phosphate-limiting conditions, e.g., human airway epithelial infections such as cystic fibrosis (Chekabab, Harel, and Dozois 2014; Jones et al., 2021). In addition, AP seems to contribute to the cell division cycle in a low-phosphate environment, possibly a consequence of its role in the inorganic phosphate scavenge (Bhatti, DeVoe, and Ingram 1976). To the best of our knowledge, there is no recent scientific literature reporting effective inhibitors to AP of organisms phylogenetically close to *P. aeruginosa*. In contrast, several inhibitors are described to mammalian AP since they have an important role in bone formation and prevention of intestinal inflammation. Bacterial and mammalian AP have significant differences regarding their catalytic sites (number and type of metal ions, amino acid residues), molecular weight, and kinetics (Rashida and Iqbal 2015). Indeed, Chakraborty et al. (2012) described the inhibition of *Vibrio* AP by imipenem, a β-lactam antibiotic, but the same effect was not observed in the *Escherichia coli* AP. Even inside the same domain, the AP of these phylogenetically distant organisms is not affected by the same compounds. Thus, it is theoretically possible to discover a drug capable of inhibiting the *P. aeruginosa* AP without an undesired effect on the host.

The gene *metG* encodes the enzyme methionyl-tRNA synthetase (MetRS), which belongs to the same class of two other targets identified in our work: *hisS* (histidyl-tRNA synthetase, HisRS) and *thrS* (threonyl-tRNA synthetase, ThrRS) (Table 1). Overall, aminoacyl-tRNA synthetases (AaRSs) constitute a class of 20 enzymes essential for protein biosynthesis, corresponding to each canonical amino acid. AaRSs catalyze a specific amino acid attachment to their cognate tRNAs, playing a crucial role during the initiation and elongation phase of protein biosynthesis. Due to their primordial function, AaRSs are present in all three kingdoms of life. Despite their similarity among organisms, structural differences between prokaryotic and eukaryotic AaRSs are sufficient to select pathogen-specific inhibitors (Kwon, Fox, and Kim 2019; Pang, Weeks, and Van Aerschot 2021). There are two known AaRSs inhibitors approved for clinical use, but none are designed for Gram-negative pathogens. Most bacteria contain one of the two forms of a MetRS, where MetRS1 is found in Gram-positive bacteria, protozoa, and mitochondria, and MetRS2 is found in archaea, the cytosol of eukaryotic cells, and Gram-negative bacteria, including *P. aeruginosa* (Nakama, Nureki, and Yokoyama 2001; Gentry et al., 2003; Rock et al., 2007). According to Mercaldi et al. (2021), the auxiliary pockets of MetRS1 and MetRS2 differ in their amino acid composition, leading to less or no effect of known MetRS1 inhibitors upon MetRS2. In *P. aeruginosa*, Robles et al. (2017) found one candidate among 1,690 compounds with satisfactory inhibition results, the isopomiferin. However, isopomiferin did not show broad-spectrum activity, in addition to high-level toxicity in human cells when compared to other antibiotics of common use. Instead, promising compounds have been found for the other two targets, HisRS and ThrRS. A screening assay analyzing nearly 1700 compounds selected 15 with activity against HisRs of *P. aeruginosa*. Among them, four (BT02C02, BT02D04, BT08E04, and BT09C11) were highlighted for presenting effective inhibition results associated with a broad-spectrum activity. Furthermore, the compounds bound to other places besides the active site of aminoacylation, which is advantageous to avoid resistance mechanisms, having low-level toxicity in eukaryotic cells. An interesting feature of BT09C11 is the presence of a sulfonamide group, which has antimicrobial activity against other enzymes, e.g., acting as a competitive inhibitor of dihydropteroate synthase (an enzyme encoded by another potential target, the gene *folP*). This fact could imply more than one form of inhibition (Henry 1943; Hu et al., 2018). Regarding ThrRS, Scott et al. (2019) described obafluorin, a natural compound produced by *Pseudomonas fluorescens*. Obafluorin is active against Gram-positive and Gram-negative bacteria, including *P. aeruginosa*. Interestingly, *P. fluorescens* has a homolog to its ThrRS called ObaO, which confers immunity to obafluorin, and it is not present in the *P. aeruginosa* chromosome.

The gene *fabB* encodes the cytoplasmic enzyme 3-oxoacyl-[acyl-carrier-protein] synthase 1 or β-ketoacyl-ACP synthase (KAS) I (Feng and Cronan 2009). In *P. aeruginosa*, *fabB* is co-transcribed with *fabA* establishing the *fabAB* operon that plays a crucial role in unsaturated fatty acid (UFA) biosynthesis via the anaerobic type II biosynthetic pathway (Hoang and Schweizer 1997; Subramanian, Rock, and Zhang 2010). The joint function of both enzymes FabA and FabB impact the membrane fluidity under different growth conditions contributing to the dominant UFA synthetic pathway in *P. aeruginosa*. Indeed, the composition of *P. aeruginosa* membrane contains more UFAs than saturated fatty acids, whose balance depends on a coordinated regulation at the transcriptional level in response to changes in the environment (Zhang et al., 2007). Two major inhibitors of KAS were described to date, cerulenin and thiolactomycin. Although *fabB* was not considered homologous to human proteins in our analysis, cerulenin was not selective also inhibiting eukaryotic KAS. In contrast, thiolactomycin interacts with FabB preventing the elongation of UFAs. It has a broad spectrum against several pathogens and is selective (Jackowski et al., 2002; Khandekar, Daines, and Lonsdale 2003). In *P. aeruginosa*, Schweizer (1998) demonstrates intrinsic resistance to thiolactomycin conferred by efflux pump systems. However, the critical role of FabB in the composition of cellular fatty acids and membrane fluidity of *P. aeruginosa* emphasizes its importance as a drug target, and the combination of existing drugs with antimicrobial adjuvants like efflux pump inhibitors could lead to effective therapeutic options (Peraman et al., 2021).

The gene *dapB* encodes the enzyme dihydrodipiconilate reductase (DHDPR). DHDPR catalyzes an intermediate reaction in the diaminopimelate (DAP) pathway responsible for the biosynthesis of two essential compounds, *meso*-diaminopimelate (*meso*-DAP) and lysine. Lysine is important for protein synthesis and *meso*-DAP is a cell wall component in Gram-negative bacteria, such as *P. aeruginosa.* In mammalians, lysine is an essential amino acid, i.e., it is not synthesized and must be acquired from the diet. DAP pathway is only present in bacteria and plants. These points reinforce the potential of DapB inhibitors as antimicrobial agents and minimize the possibility of toxicity in human cells (Hutton, Perugini, and Gerrard 2007; Impey et al., 2020). The efforts towards the identification of DHDPR inhibitors are focused on *Mycobacterium tuberculosis*. Some effective candidates were found, including sulfonamides. Among them, one has a sulfonamide group replaced by a sulfone, showing an increased potency against DHDPR of *M. tuberculosis* in addition to DHDPR of *E. coli* (Paiva et al., 2001). There are no reports in the scientific literature on investigating *dapB* for drug discovery in *P. aeruginosa*.

Although low-ranking targets, the genes *kdsA* and *rmlA* also stand out in Table 1 because they were identified as potential drug targets in all models. It is noteworthy that both genes are involved in the biosynthesis of lipopolysaccharide (LPS) constituents. LPS is a major component of *P. aeruginosa* outer membrane. It is an important virulence factor and an efficient permeability barrier. The *kdsA* gene encodes a key enzyme 2-dehydro-3-deoxyphosphooctonate aldolase that catalyzes the production of 2-keto-3-deoxy-D-manno-octulosonate-8-phosphate, an essential compound for the assembly of LPS (Nelson et al., 2013; Valvano 2015). The *kdsA* gene is part of the essential core genes described by Poulsen et al. (2019), and previous works corroborate its essentiality through experimental techniques and different media (Skurnik et al., 2013; Lee et al., 2015; Turner et al., 2015). The inhibition of KdsA leads to cell growth arrest by limiting replication (Xu et al., 2003; Ahmad et al., 2019). There are several inhibitors of KdsA described with potent *in vitro* activity, including the {[(2,2-Dihydroxy-Ethyl)-(2,3,4,5-Tetrahydroxy-6-Phosphonooxy-Hexyl)-Amino]-Methyl}-Phosphonic acid (DB02433) present in DrugBank as an experimental drug (Grison et al., 2005; Harrison, Reichau, and Parker 2012; Ahmad et al., 2019). The gene *rmlA* encodes the enzyme glucose-1-phosphate thymidylyltransferase (G1PTMT), which catalyzes the first step in the biosynthesis of rhamnose, a homopolymer component of *P. aeruginosa* LPS (King et al., 2009; Alphey et al., 2013). Poulsen et al. (2019) did not mention *rmlA* in their work, but a knockout mutant of the *rmlA* gene in PAO1 could not grow in M9 minimal medium. In addition, the mutant released very low extracellular DNA, which is related to biofilm formation and induction of antibiotic resistance in biofilm (Elamin et al., 2017). The substrates of G1PTMT are glucose-1-phosphate and deoxy-thymidine triphosphate. Smithen et al. (2015) demonstrate that bisubstrate analogs, i.e., a molecule that resembles both substrates in a transient state, are potent inhibitors of G1PTMT of *Streptococcus pneumoniae.* In *P. aeruginosa*, Alphey et al. (2013) show small thymidine-containing molecules that inhibit G1PTMT through binding the allosteric site. Allosteric inhibitors are considered more promising drugs because they are more specific thus less toxic. The reason is that allosteric sites often are more selective due to lower amino acid residue conservation among protein families when compared to active sites. Despite recent advances, the discovery of G1PTMT inhibitors to be used as antimicrobial agents remains a challenge. The genes *kdsA* and *rmlA* have numerous essential criteria for prioritization, such as essentiality, absence of homologs in humans, broad-spectrum target, and druggability. However, they did not obtain a good classification because we used the microbiome conservation criterion to rank the identified targets, minimizing adverse effects caused by the elimination of intestinal flora. However, this is not an exclusion criterion, only the parameter of prioritization adopted in our work.

The main goal in building computational models based on different layers of biological data is to improve the accuracy of *in silico* simulations. We demonstrate that the integration of transcriptome data to metabolic networks described in our work successfully achieved this objective, resulting in growth curves and flux distributions in line with biological observations. In addition, the identification of potential drug targets from integrated computational models is more selective and points out genes with reported biological relevance. Indeed, some targets identified in our work have already been proposed as drug targets through distinct methodologies. Others are already known drug targets. These observations corroborate that further investigating unexploited targets is a promising approach. A noteworthy remark is that the reference organism used in this work is not a multidrug-resistant strain. However, the methodology applied here can be extended to other strains, other genera, and other conditions since “omics” data are available for several organisms. Finally, we advocate integrating multiple layers of omics data for accurate phenotype prediction and therapeutic target identification, enabling new drug discovery through advanced systems biology approaches instead of time-consuming and expensive conventional screening.

## Data Availability

The original contributions presented in the study are included in the article/Supplementary Material, and available at https://github.com/medeirosfilho1/Integrationpaeruginosa. Further inquiries can be directed to the corresponding authors.

## Abbreviations

AaRS, aminoacyl-tRNA synthetase; AM, metabolic model with adjusted carbon source uptake rate; AP, alkaline phosphatase; DAP, diaminopimelate; DHDPR, dihydrodipiconilate reductase; FBA, flux balance analysis; FVA, flux variability analysis; G1PTMT, glucose-1-phosphate thymidylyltransferase; GEM, genome-scale metabolic model; GPR, gene-protein-reaction; HisRS, histidyl-tRNA synthetase; INT, integrated model; KAS, ketoacyl-ACP synthase; LPS, lipopolysaccharide; MALS, malate synthase; MetRS, methionyl-tRNA synthetase; ThrRS, threonyl-tRNA synthetase; UFA, unsaturated fatty acid.
